# 

**Published:** 1893-07-22

**Authors:** 


### CONTENTS.

Will Specialists Become Extinct? - 257

Around the Hospitals - 258

Medical History from the Earliest Times. By E. T. WITHINGTON, M.A., M.B.Oxon. LXIII.—The Brunonian System 259

Contemporary Theory and Research - 260

Diet in Disease. By MRS. ERNEST HART. XXVI.—On Thinning and Fattening (*continued*) - 261

The Hospital Clinic—

ROYAL HOSPITAL FOR DISEASES OF THE CHEST, CITY ROAD, E.C.—The Treatment of Cough as a Symptom in Phthisis - 265

LONDON HOSPITAL.—Treatment of Cancer of the Breast - 266

ROYAL ORTHOPÆDIC HOSPITAL.—Treatment of Stiff Joints - 267

ON A FORM OF INDIGESTION AND ITS TREATMENT. - 268

NEW APPLIANCES AND THINGS MEDICAL - 268

The History and Capabilities of Herbal Simples. By W. T. FERNIE, M.D.—Garlic and the Onion - 262

Annotations.—Cycling and Heart Disease—Cholera, Tea, and Trade—The Ups and Downs of Doctors - 263

Notes and News - 264

The Institutional Workshop—

WITHIN THE HOSPITALS.—Guy's Hospital - 269

THE STORY OF THE INSANE FROM YEAR TO YEAR - 270

PRACTICAL DEPARTMENTS.—Furniture for Nurses' Rooms - 271

FESTIVAL DINNERS AND MEETINGS - 271

CONSTRUCTION NOTES - 272

Scraps and Gleanings - 272

The Book World of Medicine and Science - vii

Medical Vacancies and Appointments - ix

### EXTRA SUPPLEMENT.—THE NURSING MIRROR.

News from the Nursing World.—A Royal Visit—A Plea for Sick Paupers—Are you Registered, Nurse?—Grievances—Where and When—What Becomes of the Flowers?—"Someone had Blunder'd"—Must the Wards be Closed?—District Nurses in America—Miss Nightingale's Paper—On Land and Sea—Male Nurses—A Noble Example—Short Items clxi

Sanitation for Nurses. By A SANITARY INSPECTOR. XIV.—Heat as a Disinfectant - clxiii

"The Pall Mall Gazette" and the London Hospital - clxiv

Everybody's Opinion - clxv

Meetings.—The R.B.N. Association - clxvi

Educational Standards for Nurses. By Miss ISABEL A. HAMPTON. II.—Present Educational Advantages and Deficiencies - clxiv

Church of England Sanitary Association - cl[illegible]vi

Where to Go - clxvi

The International Nursing Congress, Chicago. By OUR SPECIAL COMMISSIONER. III.—District Nursing - clxvi

British Columbia.—A Hospital for the Indians - clxviii

For Reading to the Sick—Trifles—Notes and Queries - clxviii

Appointments.—Minor Appointments - clxviii

The Book World for Women and Nurses - clxix

